# Impact of the intervention program “JolinchenKids – fit and healthy in daycare” on energy balance related-behaviors: results of a cluster controlled trial

**DOI:** 10.1186/s12887-019-1817-8

**Published:** 2019-11-13

**Authors:** Berit Steenbock, Christoph Buck, Hajo Zeeb, Stefan Rach, Claudia R. Pischke

**Affiliations:** 1 Leibniz Institute for Prevention Research and Epidemiology – BIPS, Achterstrasse 30, 28359 Bremen, Germany; 2 Center for Clinical Psychology and Rehabilitation, University of Bremen, Bremen, Germany; 3 Health Sciences Bremen, University of Bremen, Bremen, Germany; 4 Centre for Health and Society, Medical Faculty, Institute of Medical Sociology, Heinrich Heine University Duesseldorf, Moorenstrasse 5, 40225 Duesseldorf, Germany

**Keywords:** Preschoolers, Daycare, Health promotion, Motor skills, Nutrition, Body composition, Physical activity

## Abstract

**Background:**

The purpose of this study was to evaluate the multi-component health promotion program, *JolinchenKids – fit and healthy in daycare*, designed to promote physical activity (PA), healthy eating, and mental wellbeing among 3- to 6-year-old preschoolers.

**Methods:**

For this cluster controlled trial, 62 daycare facilities (DFs) from thirteen different federal states in Germany were recruited (31 intervention, 31 control DFs). Outcome measures were children’s habitual PA, fruits and vegetable consumption, consumption of unsweetened beverages and snacks with parents as raters. Study nurses assessed children’s body composition and motor skills. Data was collected at baseline and 12 months later. To track adherence to the implementation of intervention modules at individual DF groups, an implementation calendar was used from baseline to follow-up. Linear mixed models were used to investigate effects for survey, group and their interaction at the individual level while accounting for clustering.

**Results:**

Samples of 831 (baseline) and 641 (follow-up) children aged 4.3 ± 0.8 and 5.2 ± 0.8 years were analysed. More than half of the intervention DFs chose the nutrition or PA module for the first year of implementation while an implementation level of > 50% was only achieved in less than a third. A significant intervention effect (survey × group interaction) was found for the standing long jump favouring children at intervention DFs (β = 3.08; 95% Confidence interval [CI]: (0.09; 6.07)). No significant intervention effects were found for time spent on PA, total screen time, dietary habits, and body composition, i.e. body-mass-index and percentage of body fat.

**Conclusions:**

Participation in *JolinchenKids – fit and healthy in daycare* led to improvements in some indicators for motor skills. However, other health outcomes and behaviours were not affected by program participation over the course of 1 year.

**Trial registration:**

German Clinical Trials Register DRKS00011065 (Date of registration 16-09-2016).

## Background

Fostering young children’s mental and physiological health and examining the impact of programs for health promotion represents a major goal for researchers, policy makers and practitioners in various settings, including daycare. This entails an age-appropriate growth and development [1], including a healthy body weight and proper development of motor skills, as well as of social and emotional skills. Unhealthy body weight is a major concern in childhood because childhood overweight and obesity is accompanied by multiple negative health consequences in the short and long-term [2–7]. Evidence suggests that excessive weight gain in the first years of life increases the risk for obesity and chronic diseases, such as coronary heart disease, later in life [8–10]. In fact, obese preschool-aged children are five times more likely to be overweight during adolescence and have a four times increased risk to be obese in adulthood when compared to normal weight children [11]. Unhealthy eating and PA habits, which are known to contribute to the etiology and progression of lifestyle-related diseases, are, to a great extent, learned during the first years of life and tend to persist into childhood, adolescents, and adulthood [12–16]. In Germany, to date, 50 % of children under the age of 6 years do not meet the current PA recommendation for their age group [17]. Only one fifth of 3- to 6-year-old children consume the recommended five or more portions of fruits and vegetables per day [18]. In young children, higher levels of locomotor skills are positively associated with moderate-to-vigorous PA [19] and inversely associated with skinfold thickness and waist circumference [20]. The influence of motor development is therefore of importance to the general development of young children. Therefore, health promotion in young children should not only focus on health-related outcomes but comprehensive programs should be offered that also include components targeting motor skill development and learning [21].

To date, the impact of PA and dietary interventions in the preschool setting is frequently less than optimal and comprehensive evaluations of intervention effects are rare [22, 23]. Studies that examined the effects of single-component interventions, mostly targeting PA, to prevent or reduce childhood overweight and/or obesity demonstrated no, inconsistent, or only weak effects [24]. Therefore, the provision of interventions that target both PA and dietary habits combined is highly recommended [25]. Further, evidence suggest that the key elements of successful approaches are ‘modelling’ (through older peers, teachers and parents) and techniques to facilitate skill development among children and parents [25, 26].

This study was aimed at evaluating the effectiveness of a comprehensive health promotion program in the preschool setting after 1 year of implementation. *JolinchenKids – fit and healthy in daycare* is a multi-component program mainly targeting PA, healthy eating, and mental wellbeing in 3- to 6-year olds, which lasts for 3 years. It was originally designed by the German health insurance AOK (“Allgemeine Ortskrankenkasse”, General Local Health Insurance) and was aimed at promoting a healthy development in preschoolers through playful modification of children’s lifestyle behaviours supported by changes in the daycare environment and parental participation. In this intervention study, children participating in the program at intervention daycare facilities (DFs) were compared to those enrolled at delayed intervention control DFs receiving the intervention after the end of the study.

## Methods

### Description of ‘JolinchenKids – fit and healthy in daycare’

*JolinchenKids - fit and healthy in daycare* is comprised of five modules, three focusing on children, one on parental participation, and one on promoting health among DF staff. The three modules focusing on children’s health were designed to affect dietary and PA habits among 3- to 6-year-olds and to improve their mental well-being [27]. Program Implementation was based on the Public Health Action Circle and included the four steps: [1] needs analysis, [2] module selection, [3] module implementation, and [4] evaluation. Starting point of the program was a two-day training session for teachers followed by the needs analysis and module selection in each individual DF. During the two-day training session, staff of the AOK health insurance familiarized teachers with content and objectives of each module, explained how to use the intervention materials and gave practical tips for the selection and pedagogical and didactic implementation of the individual modules of JolinchenKids into the day-to-day routines of the daycare setting. Generally, DFs were free to choose which modules they would like to implement and in which order. In addition, several modules could be implemented at the same time. More detailed information about the program and intervention components belonging to the five different intervention modules can be found elsewhere [27].

### Study design and participants

The evaluation of *JolinchenKids – fit and healthy in daycare* was conducted with a nationwide sample of DFs. A cluster-controlled trial was conducted to assess relevant outcome measures at baseline and after 1 year. The AOK provided two lists, one containing addresses of DFs implementing the program in 2016 and one containing DFs that planned to implement the program 1 year later. We invited a random selection of DFs (*n* = 473) from both lists to participate in the study. To ensure that DFs from rural and urban parts of Germany would take place in the study, the number of inhabitants was determined for each DF on the basis of the postal code. The selection then followed a ranked list of DFs classified into quintiles based on the number of inhabitants (≤ 5.000; ≤ 20.000; ≤ 100.000; > 100.000). In the invitation letter, we provided a short questionnaire to determine eligibility to participate in the study. Further information on the short questionnaire and the inclusion criteria can be found elsewhere [27]. The recruitment took place in summer and fall 2016. DFs were closed during school holidays depending on the federal state. We started recruiting directly after the closing times. The first surveys could therefore be carried out in September. To ensure that both groups were similar in structural characteristics and in parents’ socio-demographic characteristics, information from the short questionnaire was used to match intervention and control DFs.

Once a DF consented to participate in the study, was deemed eligible, and was matched to a DF from the control group, we asked the DF to choose two kindergarten groups that included at least some three-to-five-year-old children. Parents of three-to-five-year-old children of those two groups were invited to have their child to participate in the study. Prior to the tests, parents were asked for informed consent to participate in each survey. On the measurement day, children with written parental informed consent were informed appropriately and asked for verbal assent. Ethical approval for conducting the study was obtained from the Medical Association in Bremen (HR/ RE – 522, April 28th, 2016).

Between September and December 2016, baseline measurements were performed followed by a post-test assessment during the same time period 1 year later (Sept – Dec 2017). The same two study nurses who were trained by two researchers collected data at both time points. After the baseline assessment, program implementation started at intervention DFs with a two-day training session for teachers for the introduction of the programme and distribution of intervention materials provided by the AOK. Control DFs did not receive the intervention and continued with their usual routine. Upon completion of the final data collection, the control DFs were offered the same training and materials that had been delivered to the intervention DFs (i.e. wait listed control).

### Measurements

#### Demographic information

Parental education, monthly household income, and children’s sex, age, and migration background were reported by parents (or legal guardians) [27]. If information on education was available from both parents/legal guardians, parental education was classified into low, medium, and high according to Lampert et al. [28], otherwise it was set to missing. Data on migration background was compiled based on information on the country of birth and the nationality of both parents. Children classified as having a two-sided migration background had parents who both had immigrated to Germany and/or parents who were not German citizens; children classified as having a one-sided migration background had one parent that had immigrated to Germany from another country and/or did not hold German citizenship [29]. If information on the country of birth or the nationality of one parent was not available, migration background of the child was set to missing. To determine urbanity, DFs from municipalities with more than 20.000 inhabitants were classified as urban whereas those from municipalities with less than 20.000 inhabitants were classified as rural.

#### Anthropometry

To measure height, weight, and body composition, children had to be barefoot. Height was measured to the nearest 1 cm using a stadiometer (Seca® type 213 stadiometer, Invicta Plastics Ltd., Leicester, UK). Body weight was measured to the nearest 0.1 kg. Bioelectrical impedance and body mass were assessed once in each child using a prototype leg-to-leg device that is based on the TANITA® BC 420 SMA digital scale. The prototype was developed by TANITA Europe (TANITA Europe GmbH, Sindelfingen, Germany) specifically for this study to assess leg-to-leg bioelectrical impedance in children whose feet are too small for the currently produced devices. The BIA measurements took place according to the instructions of the manufacturer [30]. Body type was entered as ‘standard’ for all children. For ethical reasons, children were measured without fasting. Before each individual measurement, the child’s shoes and socks and, if worn, tights were removed. The body mass index (BMI) was calculated as weight in kilos measured by the TANITA scale divided by body height in meters squared. We did not weigh children’s clothes; therefore, we did not correct BMI for the weight of the clothes. Children were classified as underweight/normal or overweight/obese according to age- and sex-specific cut-offs derived from percentile curves by Cole and Lobstein [31]. These (International Obesity Task Force – IOTF) childhood BMI cut-offs for overweight, obesity, and thinness are widely used and are based on nationally representative survey data from six countries covering the age range of 2 to 18 years [31]. Data were fitted using the LMS method to standardize the distribution of BMI using age- and sex-specific parameters on skewness (L), median (M), and coefficient of variation (S), respectively in the age range from 2 to 18 years to eventually fit corresponding BMI categories for adults at age 18. If information on height and weight was not available, BMI and BMI category were set to missing. Percent body fat was derived from bioelectrical impedance assessment (BIA) [32]. Due to the lack of a fasting state at the time of measurement, implausible BIA values could not be avoided. According to Goran et al. [33] values under 500 and above 900 Ω were excluded from the data analyses later on. If information on BIA was not available or in case of an implausible BIA value, percent body fat was set to missing.

#### Motor skills, screen time and physical activity

Children participated in the five test items of the KindergartenMobil-Test (KiMo). A detailed description of the testing procedure is given by Klein and colleagues [34]. Further information of the five test items can be found elsewhere [27]. Exercises were explained and demonstrated according to the KiMo manual. We analyzed items separately for each motor skill.

To assess screen time, parents were asked about information on hours of television/digital video disk/video viewing (television time) and computer/smartphone/tablet/games-console use (computer use) for both a typical weekday and weekend days. Response categories were 0 = ‘not at all’, 1=’ < 1 h/day’, 2 = ‘between 1 and < 2 h/day’, 3 = ‘between 2 and < 3 h/day’, and 4=’ > 3 h/day’. Children’s television time and computer use were summed up to total screen time per week as follows: (television time on weekdays*5) + (television time on weekend days*2) + (computer use on weekdays*5) + (computer use on weekend days*2). Daily PA levels were assessed via parental reports based on a question in the parental questionnaire: “Think for a moment about a typical weekday (weekend day) for your child in the last month. How much time would you say your child spends playing outdoors on a typical weekday (weekend day)?”. In addition, parents answered the following questions:” Is your child member of a sports club?” and “How much time does he/she spend doing sports in a sports club per week?”. Hours that children spent playing outdoors on a typical weekday and on weekend days and weekly participation in sports club activities were assessed to calculate total daily time spent on PA as follows: [(PA playing outdoors on weekdays*5) + (PA playing outdoors on weekend days*2) + weekly sports participation]/7. These parental-report measures of outdoor playtime and sports participation were found to be positively correlated with accelerometry-derived moderate to vigorous PA in a previous study [35].

#### Healthy eating

We assessed consumption of unsweetened beverages, fruits and vegetables, snacks, and the number of meals per day during the last week in a self-developed food frequency questionnaire (FFQ) which has not been validated so far. However, items were based on a validated FFQ previously used in wave 2 of the longitudinal cohort study “German Health Interview and Examination Survey for Children and Adolescents” (KiGGS) [36, 37] and food categories were based on the food pyramid of the German Nutrition Society (DGE, Deutsche Gesellschaft für Ernährung). In the self-administered FFQ, respondents are asked questions about the frequency and about the portion size of a limited number of usually consumed foods. It is relatively inexpensive, easy and quick to administrate [38]. However, only a limited number of foods can be included in a FFQ for feasibility reasons and to limit the burden for participants. Intervention goals in the nutrition module of *JolinchenKids – fit and healthy in daycare* are based on the nutrition pyramid of the AID Infoservice of the Federal Office for Agriculture and Food [39] which is based on the recommendations of the DGE. When developing the FFQ used in our study, we therefore adapted a predefined list of foods to the nutrition pyramid of the AID. Items were developed and used to measure the consumption of water and unsweetened drinks, fruits, vegetables, bread, cereals and side dishes, milk (products), meat, fish and eggs, fats and oils, and sweets, sweet spreads, pastries or salty snacks. To assess consumption of unsweetened beverages parents were asked the following “How often did your child drink water (mineral water, tap water, homemade soda water) and unsweetened drinks (fruit tea, herbal tea) in recent weeks?” Response categories were “never/ less than one glass per week”, “one to three glasses per week”, “four to six glasses per week”, “one glass a day”, “two to three glasses a day”, “four to five glasses a day”, and “more than five glasses a day” wherein one glass was defined as 135 ml. To assess number of snacks per day we asked parents the following question: “How often did your child eat sweets, sweet spreads, pastries or salty snacks such as chips and french fries in recent weeks?” Response categories were “never/ less than one serving per week”, “one to three servings per week”, “four to six servings per week”, “one serving a day”, “two servings a day”, “three servings per day”, “four servings per day”, and “more than four servings a day”. One portion size was quantified as one child’s hand full of a snack. In addition, the number of meals on weekdays and weekends was assessed. Based on the recommendations of the nutrition pyramid [39], nutrition was considered healthy, if a child consumed i) at least four glasses of unsweetened beverages, ii) at least five portions of fruits and vegetables, iii) not more than one snack per day, and iv) at least three portions of milk and dairy products per day.

#### Family health climate

The family health climate was assessed with the Family Health Climate-Scales for PA (FHC-PA) and nutrition (FHC-NU), using a validated questionnaire [40]. The FHC-PA Scale consists of 14 items and three subscales: value (e.g.,” In our family, it is normal to be physically active in our leisure time”), cohesion (e.g.,” ...we have fun doing physical activities together (e.g., bike tours, hikes)”), information (e.g.,” ... we collect information (e.g. on the internet) on physical activity and exercise”). The FHC-NU Scale is comprised of 17 items pertaining to four subscales: value (e.g.,” ... it is normal to choose healthful foods”), cohesion (e.g., “...we appreciate spending time together during meals”), communication (e.g., “...we talk about which foods are healthful”), consensus (e.g.,” ...we rarely argue about food- or diet-related matters”). The items were rated on a 4-point Likert-type scale ranging from 0 = ‘not true’ to 3 = ‘true’. Scores representing the mean of all items were calculated for the FHC-PA and FHC-NU, respectively.

#### Process evaluation data

To assess intervention dose and fidelity, intervention DFs were provided with a paper-and-pencil calendar to track implementation progress at individual DF groups from baseline to follow-up [27]. In these calendars, DF staff documented module choices, as well as module specific activities for each week covering components of intervention modules for diet (healthy breakfast buffet, “drinking oasis”, dish of fruits & vegetables, short games), PA (PA games), wellbeing (time of card game “feel good island”, short time-outs), and parental participation (newsletter, “message in a bottle”, parent-staff evening) that were marked with checkboxes, as well as a documentation of the weekly amount of time (in minutes) spent on working with the respective intervention materials.

To quantify the intervention dose for each module, we considered a time frame of 40 weeks (i.e. 1 year excluding holidays) during which modules could be conducted within the DF groups. For each module, essential components were distinguished from additional components. For example, conducting 1 hour of PA games was considered as essential and counted as one point, while any additional 10 minutes of PA games was counted as 1/6 additional points for each week. The sum of points in the PA module of all weeks was then divided by 40 to assess the percentage of adherence. Likewise, for each week, a quarter point was given for each of the four components of the diet module, half a point was given for any activity in the parental participation module, and one point was given for 1 hour of games or timeouts for the mental wellbeing module, to assess the percentage of adherence. Eventually, the percentage of adherence for all DF groups of the intervention DFs was categorized into 0%, i.e. no adherence to the respective module, 1–50% of adherence, and more than 50% of adherence. Due to the points depending on the reported duration of components, for some modules an adherence above 100% was calculated.

### Statistical analysis

Descriptive statistics, i.e. mean and standard deviation (SD) or percentage of categories were calculated for the baseline and follow-up survey. We investigated the differences in outcome variables between T0 and T1 between the intervention and control group by using linear mixed models. In case of binary outcome variables, logistic mixed models were used. We modelled fixed effects for intervention group and survey to investigate overall group and time effects, as well as an interaction of group and survey to identify the intervention effect across all intervention DFs, regardless of module choices. Due to the flexibility of mixed models, we were able to use data on participants at baseline without observations in follow-up and accounted for repeated measurements by means of a random effect on the residual side. The effect estimates for quantitative outcomes are expressed as the difference between the mean individual changes in the intervention and the mean individual changes in the control groups. The effect estimates for binary outcomes were obtained from logistic regression models and presented as odds ratios with 95% confidence intervals. All models were adjusted for sex, age, and migration background, and BMI of the children, as well as for household income, highest educational level of parents and urbanity of the DF. Distribution of residuals and model fit were assessed with regard to Q-Q plots, residual plots and the Bayesian information criterion (BIC). In a further step, all models were also stratified by migration background, BMI category, and urbanity of the DF to investigate intervention effects in subsamples, e.g. overweight/obese children, children with a one- or two-sided migration background or children from urban vs. rural areas.

In a second step, we investigated intervention effects taking module choices and module-specific intervention dose at the DF group level into account. Based on the process evaluation data, differences in outcome variables were therefore estimated depending on adherence to the respective intervention modules. The adherence categories were used considering control and intervention group at baseline as reference while investigating changes in the control group at T1, and the three categories of module adherence. Modules were chosen with regard to specific outcomes, i.e. adherence to the PA intervention module was considered for the outcome variables time spent in PA, screen time, and motor skills, adherence to the nutrition module was considered for the outcome variables on fruits and vegetables consumption and consumption of unsweetened beverages and snacks.

Significance level was set to α = 0.05. Statistical analyses were conducted using SAS 9.3 (SAS Institute Inc., Cary, North Carolina, USA) [41] and particularly the glimmix procedure to estimate linear and logistic mixed models. We did not adjust for multiple testing in the overall analyses.

## Results

### Participant characteristics

**Fig. 1:**
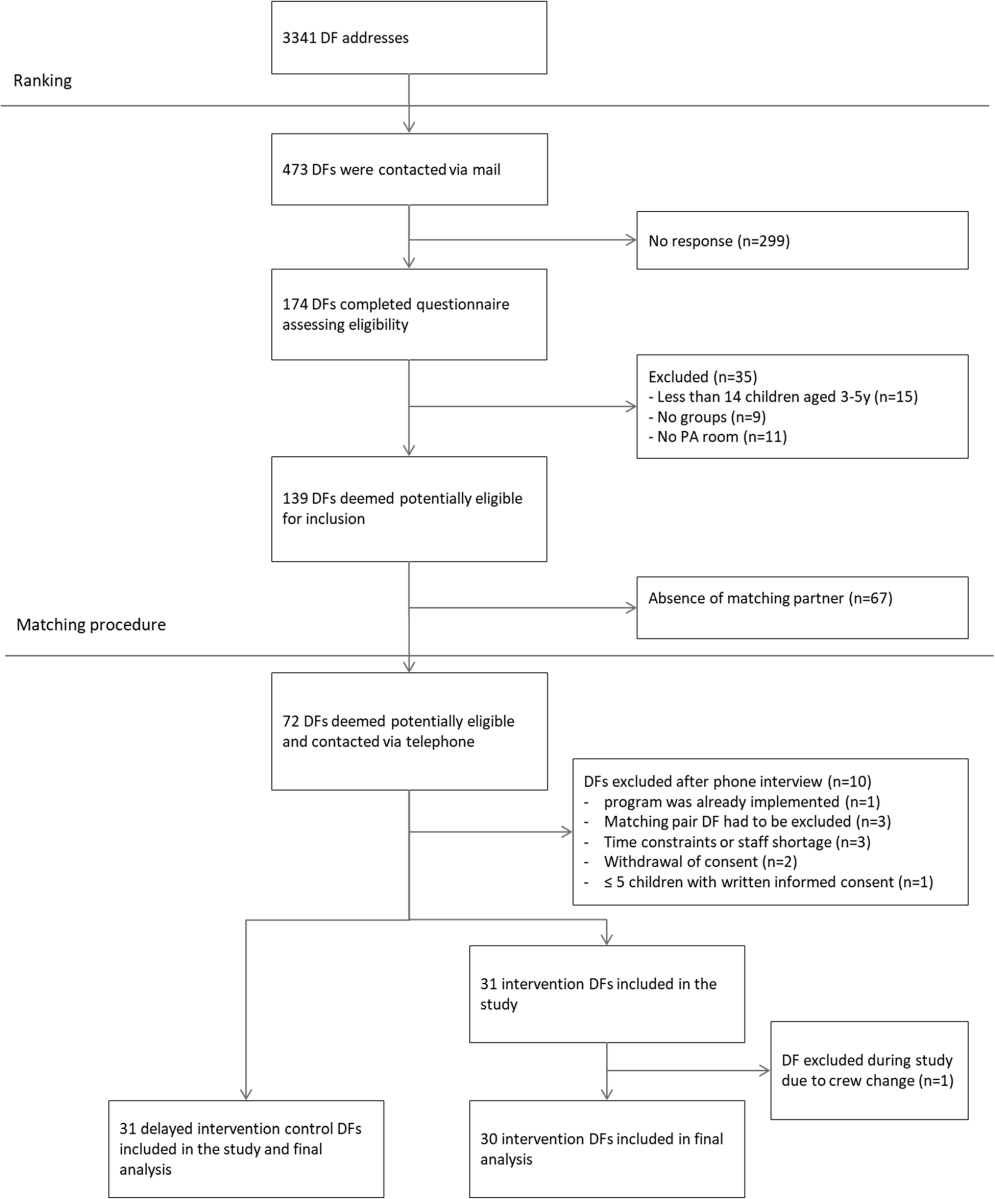
Flow diagram

One hundred seventy-four heads of DFs completed the short questionnaire assessing eligibility (response proportion: 37%) and 139 DFs were deemed eligible (80%). The matching procedure yielded a state-wide sample of 72 DFs (36 intervention, 36 control DFs). Sixty-two of the 72 DFs finally took part in the study (86%). Reasons for non-participation can be found in Fig. 1.

Descriptive characteristics of the two study groups can be found in Table 1. The mean number of children aged 3- to 5- years that were taken care of in the DF was similar in both groups (intervention group: 55,3; control group: 57,0). After exclusion of children below the age of three and above the age of 6 years, children in both groups, on average, were 4.3 years old at baseline (Table 1).

**Table 1:** Descriptive characteristics of the two study groups

	Baseline		Follow-Up	
	Intervention	Control	Intervention	Control
DFs (n)	31	31	30	31
*DFs’ characteristics (mean SD)*				
Number of children in the DF	89.7 ± 55.5	97.9 ± 50.6	–	–
Number of children aged 3- to 5- years in the DF	55.3 ± 31.0	57.0 ± 30.9	–	–
Number of DF groups	4.6 ± 2.7	5.3 ± 2.5	–	–
Percentage of children with migration background in the DF	17.5 ± 18.7	19.2 ± 22.8	–	–
Parents receiving governmental subsidies for DF fee (%) in the DF	16.3 ± 14.7	16.9 ± 20.2	–	–
Single parenting (%) in the DF	14.8 ± 13.5	13.8 ± 12.9	–	–
Children (n)	440	391	335	306
Boys (%)	50.5	51.7	49.3	53.6
Age (mean years SD)	4.3 ± 0.8	4.3 ± 0.8	5.2 ± 0.8	5.2 ± 0.8
*Migration background (%)*				
No migration background	75.9	73.9	77.6	75.8
One-sided	18.2	18.4	15.8	17.0
Two-sided	2.0	1.8	1.8	2.0
Missing	3.9	5.9	4.8	5.2
*Highest educational level of the parents (%)*				
Low	8.6	8.7	5.7	6.9
Medium	57.7	53.7	60.6	57.2
High	28.0	33.2	26.3	32.7
Missing	5.7	4.3	7.5	3.3
*Household income (%)*				
<2000€	19.1	23.3	15.5	21.9
2000–3000€	25.2	27.9	26.3	28.1
>3000€	45.2	38.9	46.6	41.5
Missing	10.5	10.0	11.6	8.5
*Urbanity (%)*				
≤5.000 inhabitants	21.4	18.9	22.7	20.9
≤20.000 inhabitants	32.5	32.7	33.1	34.6
≤100.000 inhabitants	25.2	26.6	24.5	21.6
>100.000 inhabitants	20.9	21.7	19.7	22.9
*Body-mass-index (%)*				
Underweight/normal weight	85.0	89.5	83.3	82.7
Overweight/obese	5.7	4.6	9.0	6.2
Missing	9.3	5.9	7.8	11.1
*Hours attending DF (%)*				
1–5 h/day	25.9	24.8	19.4	18.0
>5 h/day	72.7	72.1	77.3	79.7
Missing	1.4	3.1	3.3	2.3

Note: *DF* daycare facility, *SD* standard deviation

Although DFs were asked to select up to two DF groups for participation in this study, we received process evaluation data from up to five different groups per DF. In total, we received process evaluation data from 64 DF groups with outcome data ranging from one up to thirteen children per DF group. Forty-six (71.9%) of the intervention DF groups partially or fully implemented the nutrition module, 41 (64%) the PA module, 44 (68.8%) the mental well-being module, and 46 (71.9%) the module on parental participation for the first year of implementation (Table 2). Nutrition and mental well-being modules were mostly implemented with an adherence of less than 50% whereas more than half of the DF groups that opted for the PA module were implementing it with an adherence above 50%. One third of the children at intervention DF groups did not take part in the corresponding intervention modules (Table 2).

**Table 2:** Self-reported intervention dose per module

Intervention dose per module (% from 40 calendar weeks)	Children		Intervention DF groups	
	N	%	N	%
Nutrition				
Intervention adherence 0%	88	26.3	18	28.1
Intervention adherence 1 to 50%	173	51.6	36	56.3
Intervention adherence > 50%	74	22.1	10	15.6
Physical activity				
Intervention adherence 0%	113	33.7	23	35.9
Intervention adherence 1 to 50%	97	29.0	18	28.1
Intervention adherence > 50%	125	37.3	23	35.9
Mental well-being				
Intervention adherence 0%	93	27.8	20	31.3
Intervention adherence 1 to 50%	216	64.5	40	62.5
Intervention adherence > 50%	26	7.8	4	6.3
Parental participation				
Intervention adherence 0%	88	26.3	18	28.1
Intervention adherence 1 to 50%	144	43.0	29	45.3
Intervention adherence > 50%	103	30.7	17	26.6

Note: *DF* daycare facility

### Anthropometry

At baseline, 5.7% of the children of the intervention and 4.6% of the control group were either overweight or obese. At follow-up, the percentage of overweight or obese children increased to 9.0 and 6.2% in the intervention and control group, respectively (Table 3). The percentage of body fat increased from baseline to follow-up in the intervention group (9.3 to 10.5%) whereas a decrease was noted in the control group (9.4 to 9.0%) (Table 4). No significant survey × group interaction effect was found for BMI category (Odds Ratio [OR] =1.26; 95% Confidence interval [CI]: (0.66; 2.40)) and for percentage of body fat (β = 0.60; 95% CI: (0.34; 1.55)). The results of the stratified analyses can be found in Additional file 1: Tables S1-S6.

**Table 3:** Intervention effects on overweight

Characteristics	Assessment period		Time difference OR^a^ (95% CI^b^)^c^	Group difference OR (95% CI)^c^	Group-by-time interaction OR (95% CI)^c^
	Baseline	Follow-Up	Ref.: Baseline	Ref.: Control	Ref.: Control*Baseline
Overweight/ obese	%	%			
Intervention	5.7	9.0	1.38 (0.79; 2.41)	1.45 (0.65; 3.23)	1.26 (0.66; 2.40)
Control	4.6	6.2			

Note: ^a^ Odds Ratio, ^b^ Confidence interval, ^c^ Adjusted for age, gender, and migration background of the children, education and income of the parents, and urbanity

**Table 4:** Intervention effects on body-fat

Characteristics	Assessment period		Time difference β (95% CI^a^)^b^	Group difference β (95% CI)^b^	Group-by-time interaction β (95% CI)^b^
	Baseline	Follow-Up	Ref.: Baseline	Ref.: Control	Ref.: Control*Baseline
Percentage body fat	Mean (SD^c^)	Mean (SD)			
Intervention	9.3 (7.7)	10.5 (7.8)	−0.15 (− 0.88; 0.58)	0.38 (− 0.93; 1.70)	0.60 (− 0.34; 1.55)
Control	9.4 (7.5)	9.0 (7.4)			

Note: ^a^ Confidence interval, ^b^ Adjusted for age, gender, and migration background of the children, education and income of the parents, and urbanity, ^c^ standard deviation

### Motor skills, screen time and physical activity

Descriptive statistics for the five motor test items can be found in Table 5. It should be noted that shorter times in the shuttle run, as well as a lower number of contacts for the item one-leg stand represent an improvement of the performance whereas for the test items sit-and-reach, lateral jumping and standing long jump, an increase in distance or jumps is an improvement. We found a significant time effect for the shuttle run, standing long jump, lateral jumping, and one leg stand in the desired direction. This was expected because the children had grown during the study period. Children from both groups improved in these four motor skills whereas no significant time effect for the sit-and-reach task was observed (Table 5). Time spent on PA actually decreased by 12.6 min and total screen time per week increased by 1 hour when compared to baseline (Table 5). However, children at intervention DFs implementing the parental participation module did not experience these increases in screen time compared to children at intervention DFs not implementing the module (see Additional file 1: Table S5). A significant survey × group interaction effect was found for the standing long jump favouring children at intervention DFs (β = 3.08; 95% CI: (0.09; 6.07)) (Table 5). This effect was more pronounced in the subsample of children without migration background (Additional file 1: Table S1). In terms of intervention intensity, children in the group with 1–50% intervention exposure displayed a significant increase of 4.6 cm in the standing long jump and a significant decrease of 0.6 s in the shuttle run compared to the control group (Additional file 1: Table S5). No significant survey × group interaction effect was found for time spent on PA (β = − 0.33; 95% CI: (− 14.46; 13.80)) and for total screen time (β = 0.45; 95% CI: (− 0.34; 1.25)) reported by the parents (Table 5). Intervention effects stratified by urbanity are not described in further detail here but can be found in Additional file 1: Table S3.

**Table 5:** Intervention effects on motor skills and physical activity

Characteristics	Assessment period		Time difference β (95% CI^a^)^b^	Group difference β (95% CI)^b^	Group-by-time interaction β (95% CI)^b^
	Baseline	Follow-Up	Ref.: Baseline	Ref.: Control	Ref.: Control*Baseline
Shuttle run (sec)	Mean (SD^c^)	Mean (SD)			
Intervention	11.5 (2.3)	9.6 (1.4)	−1.07 (− 1.35; − 0.79)	0.26 (− 0.23; 0.75)	− 0.21 (− 0.56; 0.13)
Control	11.4 (2.5)	9.5 (1.3)			
Standing long jump (cm)					
Intervention	72.7 (21.8)	93.3 (18.4)	12.39 (10.01; 14.77)	−1.35 (−5.57; 2.87)	3.08 (0.09; 6.07)
Control	74.2 (23.8)	91.8 (20.6)			
Lateral jumping (jumps)					
Intervention	15.3 (7.5)	22.1 (7.7)	5.30 (4.36; 6.23)	−0.29 (−1.44; 0.85)	−0.30 (− 1.44; 0.85)
Control	15.5 (6.7)	22.7 (9.0)			
One leg stand (contacts)					
Intervention	10.8 (6.2)	6.0 (5.5)	−2.88 (−3.78; −1.99)	0.56 (−0.55; 1.66)	−0.55 (− 1.63; 0.52)
Control	10.7 (6.9)	5.8 (5.4)			
Sit-and-Reach (cm)					
Intervention	2.9 (4.2)	2.9 (4.3)	0.31 (−0.20; 0.83)	−0.31 (−1.11; 0.50)	−0.18 (− 0.82; 0.47)
Control	3.2 (3.9)	3.3 (4.7)			
Screen time (hours/week)					
Intervention	8 (5.7)	9 (6.1)	0.49 (−0.15; 1.13)	−0.17 (−1.17; 0.83)	0.45 (− 0.34; 1.25)
Control	8 (5.7)	9 (6.0)			
Physical activity (min/day)					
Intervention	187 (80.2)	172 (74.5)	−12.60 (−23.95; − 1.25)	9.22 (− 10.54; 28.98)	−0.33 (− 14.46; 13.80)
Control	173 (82.1)	161 (77.0)			

Note: ^a^ Confidence interval, ^b^ All models adjusted for age, gender, migration background and BMI category of the children, education and income of the parents, and urbanity, ^c^ standard deviation

### Healthy eating and family health climate

Only 12% of children at intervention and 9% at control DFs consumed the recommended five or more servings of fruits and vegetables per day at baseline compared to 13% vs. 11% at follow-up. In addition, 50% of children consumed at least four glasses of unsweetened beverages per day at intervention DFs and 49% at control DFs. At follow-up, this proportion had marginally changed to 47% at intervention and 51% at control DFs. The proportion of children that were compliant with the recommendation for snacking remained unchanged at follow-up (Table 6). Only every fifth child fulfilled the recommendation of at least three milk and milk products a day. This remained unchanged at follow-up in both groups (data not shown). Children at intervention DFs had a 66% significantly lower chance to be compliant with the recommendation for unsweetened beverages compared to controls (Table 6).

**Table 6:** Intervention effects on healthy eating

Characteristics	Assessment period		Time difference OR^a^ (95% CI^b^)^c^	Group difference OR (95% CI)^c^	Group-by-time interaction OR (95% CI)^c^
	Baseline	Follow-Up	Ref.: Baseline	Ref.: Control	Ref.: Control*Baseline
≥ 5 portions of fruits and vegetables	%	%			
Intervention	12.2	12.8	0.91 (0.52; 1.60)	1.35 (0.80; 2.28)	0.99 (0.49; 1.99)
Control	9.2	11.4			
≥ 4 glasses of unsweetened beverages					
Intervention	49.8	47.4	1.14 (0.84; 1.53)	1.07 (0.74; 1.55)	0.66 (0.45; 0.96)
Control	48.8	50.8			
≤ 1 snack					
Intervention	79.4	79.7	1.12 (0.74; 1.70)	0.81 (0.55; 1.20)	1.15 (0.68; 1.95)
Control	82.2	81.8			

Note: ^a^ Odds Ratio, ^b^ Confidence interval, ^c^ All models adjusted for age, gender, migration background and BMI category of the children, education and income of the parents, and urbanity

When comparing family health climate between both groups, the total average score for the FHC-PA was 1.3 (SD = 0.5) for baseline and follow-up (see Table 7) for children at both intervention and control DFs. The total average score for the FHC-NU was even lower with 0.8 (SD = 0.4) for children at intervention and control DFs at both time points. The family health climate remained stable over time in both groups (Table 7).

**Table 7:** Intervention effects on the family health climate

Characteristics	Assessment period		Time difference β (95% CI^a^)^b^	Group difference β (95% CI)^b^	Group-by-time interaction β (95% CI)^b^
	Baseline	Follow-Up	Ref.: Baseline	Ref.: Control	Ref.: Control*Baseline
FHC-NU total score	Mean (SD^c^)	Mean (SD)			
Intervention	0.8 (0.4)	0.8 (0.4)	0.00 (−0.05; 0.05)	0.00 (−0.07; 0.06)	− 0.02 (− 0.07; 0.04)
Control	0.8 (0.4)	0.8 (0.4)			
FHC-PA total score					
Intervention	1.3 (0.5)	1.3 (0.5)	−0.02 (−0.08; 0.04)	−0.05 (− 0.13; 0.03)	0.05 (− 0.02; 0.13)
Control	1.3 (0.5)	1.3 (0.5)			

Note: ^a^ Confidence interval, ^b^ All models adjusted for age, gender, migration background and BMI category of the children, education and income of the parents, and urbanity, ^c^ standard deviation

## Discussion

In this study, the effectiveness of a modular program for the promotion of health and health behaviour was, for the first time, evaluated in a cluster-controlled trial conducted at 62 DFs in Germany. The intervention was delivered by childcare providers at DFs with involvement of parents. To our knowledge, such comprehensive evaluations yielding high-quality evidence are still rare [23]. The characteristics of the DFs included in our study suggest that the matching of intervention and control DFs according to socio-demographic indicators appeared to have worked well. The pre−/post-comparison of health and health behaviour outcomes suggests that positive intervention effects were only noted with respect to motor skills. The intervention did not influence parental reported PA, aspects of healthy eating, and body composition of participating children over the course of 1 year.

The intervention effect on specific motor skills detected in our study which was more pronounced taking intervention-dose into account is in line with findings of a meta-analysis by Logan et al. [42]. They reported significant positive effects of motor skill interventions on fundamental movement skills in preschoolers. Contrary to our results, other studies examining the impact of nutrition and PA-related interventions in the preschool setting were able to detect small effects on eating habits and PA. To give an example, a large scale study which was conducted in six European countries, the Toybox-study, reported promising results. A greater decrease of prepacked fruit juice consumption and a larger increase of vigorous and moderate-to-vigorous PA could be demonstrated in preschoolers in the intervention group compared to a control group [43]. Another recent study from Poland found that nutrition education improved the assortment of beverages in day-care centers but, in this study, a control group was not included [44]. The absence of detectable intervention effects on energy balance related behaviours in our study can be explained by various aspects immanent to the program. DFs have 3 years, in total, to implement the five modules making up the *JolinchenKids – fit and healthy in daycare* program. During this time period, DFs are flexible in selecting the number and composition of the different modules. In addition, the selection and implementation of activities within each module can also be handled flexibly. Qualitative results stemming from focus groups with DF staff not reported here suggest that the staff appreciated the flexibility regarding the implementation of the program. Overall, we observed that the majority of intervention DFs initially selected one or two modules for the first year of implementation focusing more on topics that they were already familiar with, such as healthy eating. Here, findings of the evaluation of adherence to intervention modules suggest that approximately 50% of DFs, on average, carried out less than half of the module-specific activities during the first year of implementation. Therefore, limited or null intervention effects in this study do not imply that the program itself does not have a positive impact on children’s health and health behaviour, as it may not have been sufficiently implemented to produce positive effects. A review of 500 studies analysing the effects of interventions for primary prevention and health promotion targeting children and adolescents came to the conclusion that positive findings may be seen at an implementation level of approximately 60% [45]. Unfortunately, in our study sample, this level was only achieved in less than one third of intervention DFs over the course of 1 year.

In addition, an important characteristic of effective nutrition- and movement-related interventions is fidelity [45–47]. Some interventions are more conducive to fidelity because they are highly structured and include detailed implementation manuals but many interventions do not have these features. The manual of *JolinchenKids – fit and healthy in daycare* is very detailed and concrete instructions are provided regarding the implementation of single activities within the respective modules (e.g., via card boxes to engage children in active play). However, current intervention materials lack detailed information about which sequence of activities and dose are required for successfully implementing a module or the program as a whole and for obtaining health effects. The program was developed based on evidence-based guidelines for diet, PA [25], and mental well-being for children in this age group and we attempted to quantify implementation and health effects based on these guidelines. However, the guidelines were not met by most children over the course of the first year at the majority of DFs participating in the study and the range of implementation was probably not sufficient to promote differential health effects. The health insurance that originally developed the program is, however, very interested in using the calendar developed by the research team to track and quantify implementation in the future. Another problem is that DFs often simultaneously implement other programs [48]. This was also the case during the study period (not reported here) and might have hindered a more extensive implementation of *JolinchenKids – fit and healthy in daycare* due to time constraints in the daily routine of intervention DFs. We know from telephone interviews conducted with the heads of DFs 6 months into the intervention that approximately half of the DFs were implementing other programs during the intervention period. Those programs were targeting other (health-related) topics and/or healthy eating and physical activity. Possibly, control DFs implemented such intervention components during the intervention period but this was not assessed in our study. Each federal state in Germany has its own framework for training and education and it is not obligatory to implement health promoting programs which leads to a wide variability of program activities in the daycare setting.

We found no intervention effects on anthropometric outcomes and, to our surprise, a slightly higher overall prevalence of overweight and obesity in the intervention group at follow-up based on age- and sex-specific BMI cut-off points. These results are similar to those demonstrated by other studies, of which the majority was also not able to demonstrate intervention effects on BMI or BMI categories in preschoolers [49, 50]. With regard to PA and eating behaviours, and the family health climate, there were no intervention effects as well. The intervention as implemented appeared not to succeed in carrying knowledge about health behaviour into the family environment. According to a review by Summerbell et al. [25], the most successful interventions had strong parental components. For example, they included role modelling by parents as a key intervention component, whereas mere provision of knowledge and information through distribution of letters and newsletters to parents was not sufficient [25]. *JolinchenKids – fit and healthy in daycare* fostered parental engagement but only 23% of DFs had chosen the respective module at the beginning of the study. Results of focus groups (not shown here) suggested that DF staff found it difficult to engage parents in the program, particularly if they were working full-time and had little time to contribute to the implementation of parent activities. In addition, in a previous study piloting the intervention, DFs implementing this module complained about a low parental engagement [48]. Wasenius and colleagues [51] analyzed the effect of the Activity Begins in Childhood (ABC)-intervention on fundamental motor skills in preschoolers and reported that their intervention was equally effective in increasing locomotor skills, with and without the addition of a parent-driven home PA component. They hypothesized that this finding was due to insufficient parental engagement in the intervention. Interestingly, media consumption seemed to be influenced by parental participation in the program in our study suggesting that parents influenced the behaviour of their children at home when they took part in daycare-based programs. In general, a lack of time may restrict parental engagement and whether parental participation, in general, adds benefit to health promotion in the daycare setting warrants further investigation.

Several strengths and limitations of this study need to be considered. We conducted a controlled study with a large sample across Germany, accounting for multiple confounders. However, the flexibility of the intervention enabled different module choices in the respective intervention DFs, so our sample size may not have been sufficient for testing different components. Other ongoing programs addressing partly similar topics could not be considered fully. Besides that, behavioural effects (e.g. dietary habits) in young children were difficult to assess. They were mostly based on parental report and may have been subject to reporting bias. Nutritional behaviour of children was only assessed via parental report with a FFQ that was not validated before and may represent a source of bias limiting the interpretation of our results. Furthermore, parental reports of children’s food intake lack knowledge about potential differences between dietary habits at the respective DF and at home. It is assumed that this report also includes information about meals children consumed at daycare and told their parents about at home. However, children may differ in the degree that they report at home which meals were consumed at daycare. Therefore, we may have missed favourable changes in the consumption of meals at intervention DFs. As a result, in our study, the use of a parental proxy might have led to an underestimation of intervention effects of the nutrition module. Another limitation is that we did not correct for the weight of children’s clothes and that children had not fasted before assessing their body composition.

In terms of sociodemographic characteristics, our sample was representative of children attending daycare in Germany. For example, the percentage of children with migration background in our study was 20% compared to 28% reported for children attending daycare in Germany in 2017 [52]. The overall proportion of children with a migration background in that age group, including children who do and do not attend daycare, is, however; higher (40%, 2018) [53]. In terms of weight status, the prevalence of overweight and obesity was relatively low in our sample whereas the prevalence of overweight and obesity in German children below the age of 10 years, according to data from a large study, in comparison is higher (16.5%) [54], suggesting some selection bias in the participation at the individual level*.*

## Conclusions

Participation in *JolinchenKids – fit and healthy in daycare* led to improvements in some indicators for motor skills. However, other health outcomes and behaviours were not affected by program participation, which may be partly due to a lack of sufficient implementation of the various intervention modules. Nevertheless, several single findings suggested that if individual modules were implemented with a > 50% dose, intervention effects became visible. To conclude, flexibility fosters a high program acceptance among stakeholders in the daycare setting but possibly at the expense of the effectiveness of the program. Thus, a combination of additional modules with those continuously implemented beyond the 1 year of assessment may yield synergistic effects on preschoolers’ health. Rigorous mixed-method evaluations of complex, multi-component intervention programs at DFs remain challenging, but are needed to inform program development and implementation.

## Supplementary information

**Additional file 1: Table S1.** Intervention effect based on linear mixed models, stratified by migration background. **Table S2.** Intervention effect based on logistic mixed models, stratified by migration background. **Table S3**. Intervention effect based on linear mixed models, stratified by urbanity. **Table S4.** Intervention effect based on logistic mixed models, stratified by urbanity. **Table S5**. Intervention effect based on linear mixed models, stratified by intervention dose. **Table S6.** Intervention effect based on logistic mixed models, stratified by intervention dose.

## Data Availability

The datasets used and/or analysed during the current study are available from the corresponding author on reasonable request.

## Abbreviations

AOK: Allgemeine Ortskrankenkasse (General Local Health Insurance)
BF: body fat
BMI: body-mass-index
CI: confidence interval
DF: daycare facility
DGE: Deutsche Gesellschaft für Ernährung (German Nutrition Society)
LJ: lateral jumping
LS: one leg stand
PA: physical activity
SAR: sit and reach
SD: standard deviation
SLJ: standing long jump
SR: shuttle run
