# Supplementary material for: Impact of the intervention program “JolinchenKids – fit and healthy in daycare” on energy balance related-behaviors: results of a cluster controlled trial
Source: BMC Pediatr. 2019 Nov 13;19:432. doi: 10.1186/s12887-019-1817-8 (PMC6852984; doi:10.1186/s12887-019-1817-8)
Supplement: Supplementary file 1 — Additional file 1: Table S1. Intervention effect based on linear mixed models, stratified by migration background. Table S2. Intervention effect based on logistic mixed models, stratified by migration background. Table S3. Intervention effect based on linear mixed models, stratified by urbanity. Table S4. Intervention effect based on logistic mixed models, stratified by urbanity. Table S5. Intervention effect based on linear mixed models, stratified by intervention dose. Table S6. Intervention effect based on logistic mixed models, stratified by intervention dose. [file 12887_2019_1817_MOESM1_ESM.docx]

Additional file 1: Table S1 Intervention effect based on linear mixed models, stratified by migration background.

|  | Migration background | | | No migration background | | |
| --- | --- | --- | --- | --- | --- | --- |
| Characteristics | Time difference  β (95% CI ^a^)^b^ | Group difference β (95% CI)^b^ | Group-by-time interaction  β (95% CI)^b^ | Time difference  β (95% CI)^b^ | Group difference β (95% CI)^b^ | Group-by-time interaction  β (95% CI)^b^ |
|  | Ref.: Baseline | Ref.: Control | Ref.: Control*Baseline | Ref.: Baseline | Ref.: Control | Ref.: Control*Baseline |
| **Percentage body fat** |  |  |  |  |  |  |
| Intervention | 0.72 (-1.02; 2.47) | **3.20 (0.40; 5.99)** | -1.08 (-3.50; 1.33) | -0.35 (-1.15; 0.44) | -0.51 (-1.85; 0.83) | 0.95 (-0.07; 1.96) |
| Control |  |  |  |  |  |  |
| **Shuttle run (sec)** |  |  |  |  |  |  |
| Intervention | **-1.21 (-1.85; -0.57)** | 0.38 (-0.29; 1.04) | -0.06 (-0.90; 0.78) | **-1.04 (-1.36; -0.72)** | 0.28 (-0.25; 0.81) | -0.28 (-0.66; 0.11) |
| Control |  |  |  |  |  |  |
| **Standing long jump (cm)** |  |  |  |  |  |  |
| Intervention | **14.17 (8.88; 19.46)** | 1.72 (-5.50; 8.94) | 0.64 (-6.44; 7.71) | **12.01 (9.35; 14.67)** | -2.16 (-6.73; 2.40) | **3.71 (0.42; 7.00)** |
| Control |  |  |  |  |  |  |
| **Lateral jumping (jumps)** |  |  |  |  |  |  |
| Intervention | 3.24 (-1.10; 5.38) | -1.02 (-3.87; 1.83) | 1.81 (-1.05; 4.66) | **5.82 (4.79; 6.84)** | -0.06 (-1.67; 1.55) | -0.83 (-2.06; 0.40) |
| Control |  |  |  |  |  |  |
| **One leg stand (contacts)** |  |  |  |  |  |  |
| Intervention |  |  |  |  |  |  |
| Control |  |  |  |  |  |  |
| **Sit-and-Reach (cm)** |  |  |  |  |  |  |
| Intervention | 0.21 (-0.99; 0.45) | -0.04 (-1.82; 1.74) | 0.17 (-1.23; 1.58) | 0.35 (-0.20; 0.90) | -0.32 (-1.13; 0.49) | -0.27 (-0.99; 0.45) |
| Control |  |  |  |  |  |  |
| **Screen time (hours/week)** |  |  |  |  |  |  |
| Intervention | 0.99 (-0.87; 2.84) | 0.84 (-1.56; 3.23) | -0.67 (-3.06; 1.72) | 0.41 (-0.25; 1.07) | -0.36 (-1.43; 0.71) | 0.77 (-0.04; 1.57) |
| Control |  |  |  |  |  |  |
| **Physical activity (min/day)** |  |  |  |  |  |  |
| Intervention | -15.51 (-44.44; 13.42) | -9.26 (-42.25; 23.73) | 14.52 (-20.81; 49.85) | **-12.44 (-24.81; -0.07)** | 15.36 (-6.60; 37.31) | -3.88 (-19.33; 11.57) |
| Control |  |  |  |  |  |  |
| **FHC-NU total score** |  |  |  |  |  |  |
| Intervention | 0.05 (-0.08; 0.18) | -0.00 (-0.16; 0.16) | 0.10 (-0.24; 0.04) | -0.01 (-0.06; 0.04) | -0.01 (-0.08; 0.06) | 0.00 (-0.06; 0.06) |
| Control |  |  |  |  |  |  |
| **FHC-PA total score** |  |  |  |  |  |  |
| Intervention |  |  |  |  |  |  |
| Control |  |  |  |  |  |  |
| **Psychological problems (SDQ)** |  |  |  |  |  |  |
| Intervention | 0.50 (-0.58; 1.59) | **1.74 (0.14; 3.33)** | -1.11 (-2.33; 0.11) | **-0.80 (-1.37; -0.23)** | 0.74 (-0.20; 1.68) | -0.29 (-1.00; 0.43) |
| Control |  |  |  |  |  |  |
| **KINDL total score (HRQoL)** |  |  |  |  |  |  |
| Intervention | -1.36 (-3.31; 0.58) | **-3.27 (-5.57; -0.97)** | 0.79 (-1.71; 3.29) | 0.14 (-0.91; 1.20) | 0.14 (-1.38; 1.66) | 0.04 (-1.28; 1.37) |
| Control |  |  |  |  |  |  |

Note: FHC-NU: family health climate scale for nutrition. FHC-PA: family health climate scale for physical activity. HRQol: health-related quality of life. SDQ: strengths and difficulties questionnaire.

^a^ Confidence interval, ^b^ All models adjusted for age, gender, migration background and BMI category* of the children, education and income of the parents, and urbanity. (*not for percentage of body-fat)

Table S2 Intervention effect based on logistic mixed models, stratified by migration background.

|  | Migration background | | | No migration background | | |
| --- | --- | --- | --- | --- | --- | --- |
| Characteristics | Time difference  OR^a^ (95% CI^b^)^c^ | Group difference OR (95% CI)^c^ | Group-by-time interaction  OR (95% CI)^c^ | Time difference  OR (95% CI)^c^ | Group difference OR (95% CI)^c^ | Group-by-time interaction  OR (95% CI)^c^ |
|  | Ref.: Baseline | Ref.: Control | Ref.: Control*Baseline | Ref.: Baseline | Ref.: Control | Ref.: Control*Baseline |
| **BMI category** |  |  |  |  |  |  |
| Intervention |  |  |  |  |  |  |
| Control |  |  |  |  |  |  |
| **≥ 5 portions of fruits and vegetables** |  |  |  |  |  |  |
| Intervention | 2.52 (0.91; 7.03) | 1.17 (0.35; 3.87) | 0.36 (0.09; 1.40) | 0.60 (0.30; 1.21) | 1.36 (0.78; 2.38) | 1.50 (0.64; 3.48) |
| Control |  |  |  |  |  |  |
| **≥ 4 glasses of unsweetened beverages** |  |  |  |  |  |  |
| Intervention | 0.72 (0.30; 1.70) | 0.62 (0.28; 1.37) | 0.66 (0.22; 1.93) | 1.20 (0.88; 1.65) | 1.19 (0.80; 1.78) | 0.67 (0.45; 1.00) |
| Control |  |  |  |  |  |  |
| **≤ 1 snack** |  |  |  |  |  |  |
| Intervention | 1.76 (0.60; 5.17) | 1.23 (0.44; 3.39) | 0.91 (0.23; 3.53) | 1.02 (0.64; 1.62) | 0.75 (0.48; 1.16) | 1.19 (0.66; 2.13) |
| Control |  |  |  |  |  |  |

Note: ^a^ Odds Ratio, ^b^ Confidence interval, ^c^ All models adjusted for age, gender, migration background and BMI category* of the children, education and income of the parents, and urbanity. (*not for BMI category)

Table S3 Intervention effect based on linear mixed models, stratified by urbanity.

|  | Rural < 20.000 inhabitants | | | Urban > 20.000 inhabitants | | |
| --- | --- | --- | --- | --- | --- | --- |
| Characteristics | Time difference  β (95% CI ^a^)^b^ | Group difference β (95% CI)^b^ | Group-by-time interaction  β (95% CI)^b^ | Time difference  β (95% CI)^b^ | Group difference β (95% CI)^b^ | Group-by-time interaction  β (95% CI)^b^ |
|  | Ref.: Baseline | Ref.: Control | Ref.: Control*Baseline | Ref.: Baseline | Ref.: Control | Ref.: Control*Baseline |
| **Percentage body fat** |  |  |  |  |  |  |
| Intervention | 0.04 (-0.80; 0.89) | 0.33 (-1.29; 1.95) | 0.89 (-0.20; 1.98) | -0.41 (-1.72; 0.90) | 0.35 (-1.84; 2.54) | 0.15 (-1.58; 1.89) |
| Control |  |  |  |  |  |  |
| **Shuttle run (sec)** |  |  |  |  |  |  |
| Intervention | **-1.00 (-1.35; -0.64)** | 0.52 (-0.16; 1.20) | -0.32 (-0.74; 0.10) | **-1.22 (-1.66; -0.77)** | 0.07 (-0.65; 0.79) | -0.09 (-0.64; 0.47) |
| Control |  |  |  |  |  |  |
| **Standing long jump (cm)** |  |  |  |  |  |  |
| Intervention | **12.12 (9.18; 15.06)** | -2.96 (-9.04; 3.67) | **4.64 (0.73; 8.55)** | **12.93 (9.05; 16.81)** | 0.34 (-5.32; 6.00) | 1.16 (-3.45; 5.78) |
| Control |  |  |  |  |  |  |
| **Lateral jumping (jumps)** |  |  |  |  |  |  |
| Intervention | **6.29 (5.04; 7.55)** | 0.12 (-1.86; 2.10) | -1.14 (-2.76; 0.48) | **4.15 (2.79; 5.51)** | -0.86 (-3.09; 1.37) | 0.82 (-0.74; 2.38) |
| Control |  |  |  |  |  |  |
| **One leg stand (contacts)** |  |  |  |  |  |  |
| Intervention | **-3.30 (-4.49; -2.11)** | -0.23 (-1.88; 1.41) | -0.33 (-1.80; 1.14) | **-2.42 (-3.72; -1.12)** | 1.23 (-0.19; 2.65) | -0.68 (-2.21; 0.85) |
| Control |  |  |  |  |  |  |
| **Sit-and-Reach (cm)** |  |  |  |  |  |  |
| Intervention | 0.28 (-0.40; 0.97) | 0.19 (-0.85; 1.23) | -0.52 (-1.37; 0.33) | 0.34 (-0.43; 1.11) | -0.81 (-2.10; 0.48) | 0.18 (-0.80; 1.17) |
| Control |  |  |  |  |  |  |
| **Screen time (hours/week)** |  |  |  |  |  |  |
| Intervention | **1.19 (0.32; 2.07)** | -0.64 (-2.00; 0.73) | 0.20 (-0.91; 1.31) | -0.45 (-1.36; 0.46) | 0.20 (-1.23; 1.63) | 0.83 (-0.28; 1.93) |
| Control |  |  |  |  |  |  |
| **Physical activity (min/day)** |  |  |  |  |  |  |
| Intervention | **-15.03 (-29.47; -0.59)** | 13.29 (-10.09; 36.66) | -2.91 (-21.39; 15.56) | -8.03 (-26.29; 10.23) | 3.58 (-29.88; 37.03) | 0.95 (-21.04; 23.04) |
| Control |  |  |  |  |  |  |
| **FHC-NU total score** |  |  |  |  |  |  |
| Intervention | -0.02 (-0.07; 0.04) | -0.05 (-0.13; 0.04) | 0.00 (-0.07; 0.08) | 0.02 (-0.06; 0.09) | 0.02 (-0.08; 0.13) | -0.03 (-0.13; 0.06) |
| Control |  |  |  |  |  |  |
| **FHC-PA total score** |  |  |  |  |  |  |
| Intervention |  |  |  |  |  |  |
| Control |  |  |  |  |  |  |
| **Psychological problems (SDQ)** |  |  |  |  |  |  |
| Intervention | -0.45 (-1.12; 0.22) | 0.59 (-0.67; 1.85) | -0.49 (-1.34; 0.36) | **-0.76 (-1.56; -0.02)** | **1.07 (0.01; 2.12)** | -0.34 (-1.26; 0.58) |
| Control |  |  |  |  |  |  |
| **KINDL total score (HRQoL)** |  |  |  |  |  |  |
| Intervention | 0.50 (-0.78; 1.77) | 0.41 (-1.62; 2.43) | -0.66 (-2.28; 0.95) | -0.96 (-2.31; 0.39) | -1.33 (-2.93; 0.26) | 1.30 (-0.38; 2.97) |
| Control |  |  |  |  |  |  |

Note: FHC-NU: family health climate scale for nutrition. FHC-PA: family health climate scale for physical activity. HRQol: health-related quality of life. SDQ: strengths and difficulties questionnaire.

^a^ Confidence interval, ^b^ All models adjusted for age, gender, migration background and BMI category* of the children, education and income of the parents, and urbanity. (*not for percentage of body-fat)

Table S4 Intervention effect based on linear mixed models, stratified by urbanity.

|  | Rural < 20.000 inhabitants | | | Urban > 20.000 inhabitants | | |
| --- | --- | --- | --- | --- | --- | --- |
| Characteristics | Time difference  OR^a^ (95% CI^b^)^c^ | Group difference OR (95% CI)^c^ | Group-by-time interaction  OR (95% CI)^c^ | Time difference  OR (95% CI)^c^ | Group difference OR (95% CI)^c^ | Group-by-time interaction  OR (95% CI)^c^ |
|  | Ref.: Baseline | Ref.: Control | Ref.: Control*Baseline | Ref.: Baseline | Ref.: Control | Ref.: Control*Baseline |
| **BMI category** |  |  |  |  |  |  |
| Intervention |  |  |  |  |  |  |
| Control |  |  |  |  |  |  |
| **≥ 5 portions of fruits and vegetables** |  |  |  |  |  |  |
| Intervention | 1.09 (0.51; 2.30) | 1.58 (0.78; 3.18) | 0.97 (0.39; 2.39) | 0.74 (0.31; 1.76) | 1.04 (0.45; 2.40) | 1.09 (0.35; 3.38) |
| Control |  |  |  |  |  |  |
| **≥ 4 glasses of unsweetened beverages** |  |  |  |  |  |  |
| Intervention | 0.98 (0.66; 1.45) | 1.39 (0.85; 2.28) | 0.70 (0.43; 1.16) | 1.35 (0.83; 2.20) | 0.76 (0.44; 1.32) | 0.58 (0.32; 1.07) |
| Control |  |  |  |  |  |  |
| **≤ 1 snack** |  |  |  |  |  |  |
| Intervention | 0.95 (0.55; 1.66) | 0.90 (0.52; 1.56) | 1.66 (0.80; 3.41) | 1.42 (0.74; 2.73) | 0.78 (0.43; 1.42) | 0.72 (0.32; 1.61) |
| Control |  |  |  |  |  |  |

Note: ^a^ Odds Ratio, ^b^ Confidence interval, ^c^ All models adjusted for age, gender, migration background and BMI category* of the children, education and income of the parents, and urbanity. (*not for BMI category)

Table S5 Intervention effect based on linear mixed models, stratified by intervention dose.

| Characteristics | Time difference  β (95% CI^a^)^b^ | Group difference β (95% CI)^b^ | Group-by-time interaction  β (95% CI)^b^ |
| --- | --- | --- | --- |
|  | Ref.: Baseline | Ref.: Control | Ref.: Control*Baseline |
| **Percentage body fat** |  |  |  |
| Module PA > 50% | -0.16 (-0.89; 0.57) | 0.38 (-0.92; 1.68) | 0.29 (-0.93; 1.52) |
| Module PA 1-50% |  |  | 1.07 (-0.30; 2.43) |
| Module PA 0% |  |  | 0.57 (-0.78; 1.91) |
| Control |  |  |  |
| **Shuttle run (sec)** |  |  |  |
| Module PA > 50% | **-1.07 (-1.36; -0.79)** | 0.26 (-0.23; 0.76) | 0.07 (-0.36; 0.50) |
| Module PA 1-50% |  |  | **-0.58 (-1.05; -0.10)** |
| Module PA 0% |  |  | -0.25 (-0.72; 0.22) |
| Control |  |  |  |
| **Standing long jump (cm)** |  |  |  |
| Module PA > 50% | **12.40 (10.02; 14.78)** | -1.35 (-5.56; 2.86) | 3.40 (-0.40; 7.21) |
| Module PA 1-50% |  |  | **4.58 (0.33; 8.83)** |
| Module PA 0% |  |  | 1.23 (-2.98; 5.43) |
| Control |  |  |  |
| **Lateral jumping (jumps)** |  |  |  |
| Module PA > 50% | **5.30 (4.37; 6.24)** | -0.29 (-1.76; 1.19) | 0.56 (-1.78; 1.00) |
| Module PA 1-50% |  |  | 0.56 (-1.03; 2.16) |
| Module PA 0% |  |  | -0.95 (-2.53; 0.64) |
| Control |  |  |  |
| **One leg stand (contacts)** |  |  |  |
| Module PA > 50% | **-2.91 (-3.80; -2.02)** | 0.55 (-0.55; 1.65) | -1.28 (-2.57; 0.02) |
| Module PA 1-50% |  |  | -0.72 (-2.15; 0.72) |
| Module PA 0% |  |  | 0.56 (-0.85; 1.97) |
| Control |  |  |  |
| **Sit-and-Reach (cm)** |  |  |  |
| Module PA > 50% | 0.31 (-0.20; 0.82) | -0.31 (-1.12; 0.50) | -0.39 (-1.22; 0.43) |
| Module PA 1-50% |  |  | 0.02 (-0.91; 0.95) |
| Module PA 0% |  |  | -0.08 (-0.99; 0.83) |
| Control |  |  |  |
| **Screen time (hours/week)** |  |  |  |
| Module PP > 50% | 0.48 (-0.16; 1.12) | -0.17 (-1.18; 0.83) | 0.30 (-0.82; 1.42) |
| Module PP 1-50% |  |  | 0.08 (-0.90; 1.06) |
| Module PP 0% |  |  | **1.41 (0.16; 2.66)** |
| Control |  |  |  |
| **Physical activity (min/day)** |  |  |  |
| Module PA > 50% | **-12.63 (-24.00; -1.26)** | 9.28 (-10.46; 29.02) | 3.69 (-13.91; 21.28) |
| Module PA 1-50% |  |  | -15.28 (-34.81; 4.25) |
| Module PA 0% |  |  | 8.65 (-10.82; 28.12) |
| Control |  |  |  |
| **FHC-NU total score** |  |  |  |
| Module Nutrition > 50% | -0.00 (-0.05; 0.05) | -0.01 (-0.07; 0.06) | -0.01 (-0.08; 0.07) |
| Module Nutrition 1-50% |  |  | 0.02 (-0.05; 0.09) |
| Module Nutrition 0% |  |  | -0.10 (-0.18; -0.01) |
| Control |  |  |  |
| **FHC-PA total score** |  |  |  |
| Module PA > 50% | -0.02 (-0.08; 0.04) | -0.05 (-0.13; 0.03) | 0.07 (-0.03; 0.17) |
| Module PA 1-50% |  |  | 0.07 (-0.02; 0.16) |
| Module PA 0% |  |  | -0.01 (-0.12; 0.10) |
| Control |  |  |  |
| **Psychological problems (SDQ)** |  |  |  |
| Module mental well-being > 50% | **-0.58 (-1.08; -0.07)** | **0.85 (0.02; 1.68)** | **-1.87 (-3.34; -0.41)** |
| Module mental well-being 1-50% |  |  | -0.27 (-0.95; 0.41) |
| Module mental well-being 0% |  |  | -0.35 (-1.30; 0.61) |
| Control |  |  |  |
| **KINDL total score (HRQoL)** |  |  |  |
| Module mental well-being > 50% | -0.13 (-1.06; 0.81) | -0.48 (-1.76; 0.80) | 0.78 (-1.90; 3.47) |
| Module mental well-being 1-50% |  |  | -0.16 (-1.45; 1.13) |
| Module mental well-being 0% |  |  | 0.90 (-0.87; 2.68) |
| Control |  |  |  |

Note: FHC-NU: family health climate scale for nutrition. FHC-PA: family health climate scale for physical activity. HRQol: health-related quality of life. PA: physical activity. PP: parental participation. SDQ: strengths and difficulties questionnaire.

^a^ Confidence interval, ^b^ All models adjusted for age, gender, migration background and BMI category* of the children, education and income of the parents, and urbanity. (*not for percentage of body-fat)

Table S6 Intervention effect based on logistic mixed models, stratified by intervention dose.

| Characteristics | Time difference  OR^a^ (95% CI^b^)^c^ | Group difference OR (95% CI)^c^ | Group-by-time interaction  OR (95% CI)^c^ |
| --- | --- | --- | --- |
|  | Ref.: Baseline | Ref.: Control | Ref.: Control*Baseline |
| **BMI category** |  |  |  |
| Module mental well-being > 50% | 1.38 (0.79; 2.43) | 1.43 (0.64; 3.20) | 0.60 (0.13; 2.71) |
| Module mental well-being 1-50% |  |  | 1.27 (0.64; 2.54) |
| Module mental well-being 0% |  |  | 1.52 (0.64; 3.63) |
| Control |  |  |  |
| **≥ 5 portions of fruits and vegetables** |  |  |  |
| Module Nutrition > 50% | 0.91 (0.52; 1.60) | 1.35 (0.79; 2.29) | 1.12 (0.42; 2.97) |
| Module Nutrition 1-50% |  |  | 0.76 (0.34; 1.73) |
| Module Nutrition 0% |  |  | 1.42 (0.57; 3.55) |
| Control |  |  |  |
| **≥ 4 glasses of unsweetened beverages** |  |  |  |
| Module Nutrition > 50% | 1.14 (0.85; 1.54) | 1.07 (0.75; 1.55) | 0.80 (0.45; 1.43) |
| Module Nutrition 1-50% |  |  | 0.69 (0.45; 1.07) |
| Module Nutrition 0% |  |  | **0.48 (0.27; 0.85)** |
| Control |  |  |  |
| **≤ 1 snack** |  |  |  |
| Module Nutrition > 50% | 1.12 (0.74; 1.70) | 0.81 (0.55; 1.20) | 1.32 (0.60; 2.90) |
| Module Nutrition 1-50% |  |  | 1.06 (0.59; 1.91) |
| Module Nutrition 0% |  |  | 1.22 (0.57; 2.64) |
| Control |  |  |  |

Note: ^a^ Odds Ratio, ^b^ Confidence interval, ^c^ All models adjusted for age, gender, migration background and BMI category* of the children, education and income of the parents, and urbanity. (*not for BMI category)
